# Azathioprine therapy for steroid-resistant Henoch-Schönlein purpura: a report of 6 cases

**DOI:** 10.1186/s12969-016-0100-x

**Published:** 2016-06-23

**Authors:** Lampros Fotis, Paul V. Tuttle, Kevin W. Baszis, Peri H. Pepmueller, Terry L. Moore, Andrew J. White

**Affiliations:** Department of Pediatrics, Division of Rheumatology, Washington University School of Medicine, One Children’s Place, Campus Box 8116, St. Louis, MO 63110 USA; Department of Internal Medicine, Division of Rheumatology and Pediatric Rheumatology, Saint Louis University School of Medicine, 1402 S Grand Blvd., Doisy Hall, Room R213A, St. Louis, MO 63104 USA

**Keywords:** Purpura, Schoenlein-Henoch, Azathioprine, Steroids, Child

## Abstract

**Background:**

A small percentage of children with Henoch-Schönlein purpura (HSP) develop a chronic form of the disease that often requires prolonged corticosteroid therapy. Disease modifying anti-rheumatic agents (DMARDs) or biologics have been successfully used to treat those refractory cases. Azathioprine is a DMARD that has been reported to be effective in HSP nephritis and in adult cutaneous leukocytoclastic vasculitis, a condition with cutaneous histology similar to HSP.

**Case presentation:**

A description of 6 cases with relapsing HSP without significant renal involvement, treated with azathioprine are reported. All 6 cases met the classification criteria for the diagnosis of HSP, had relapsing symptoms despite corticosteroid use, were successfully treated with azathioprine and were tapered off of corticosteroids. The duration of azathioprine therapy ranged from 7–21 months and no adverse events were reported.

**Conclusions:**

Azathioprine is effective in controlling prolonged relapsing symptoms of HSP, allowing earlier discontinuation of corticosteroids. This report shows that azathioprine can be included in the therapeutic options for relapsing HSP and is the first case series in the literature of azathioprine use in HSP without significant renal involvement.

## Background

Henoch-Schönlein purpura (HSP) is the most common form of systemic vasculitis in children. The peak incidence is 3 to 10 years of age. The disease is characterized by the tetrad of palpable purpura, arthritis/arthralgia, abdominal pain, and renal manifestations. Most cases recover spontaneously, and care is usually supportive, including bed rest, hydration, and pain relief with nonsteroidal anti-inflammatory drugs (NSAIDs) or acetaminophen [1]. Although corticosteroid use is controversial, it may result in accelerated resolution of symptoms; however, long-term use is usually limited due to adverse events [2]. Rarely, a subset of these patients has persistent rash, arthritis or abdominal involvement despite treatment with glucocorticoids or has recurrent disease during glucocorticoid tapering. There is a paucity of literature regarding therapy of such recalcitrant patients without renal involvement, although there are a handful of case reports using methotrexate, mycophenolate mofetil, rituximab, azathioprine, colchicine and dapsone [3–11]. In this case series, we report the successful use of azathioprine as a disease modulator in 6 patients (Table 1) with recalcitrant disease, without significant renal involvement, from two academic medical centers.

**Table 1:** Six patients with Henoch-Schönlein purpura treated with azathioprine

	Demographics	Clinical manifestations	Duration of disease prior to starting azathioprine	Azathioprine dose and duration	Glucocorticoid dose and duration	Length of remission off all medication
Patient 1	7 y/o female	Rash, abdominal pain	5 weeks	1 mg/kg/day for 11 months	Discharged on methylprednisolone 1 mg/kg/day tapered over 6 weeks (total of 9 weeks including 3 weeks of inpatient treatment)	15 months
Patient 2	11 y/o female	Rash, arthralgias, abdominal pain	7 months	1 mg/kg/day for 7 months	Prednisone 0.5 mg/kg/day and taper over 6 weeks	8 months
Patient 3	8 y/o female	Rash, arthralgias	5 months	1 mg/kg/day for 13 months	Prednisone 0.5 mg/kg/day and taper over 6 weeks	4 years
Patient 4	9 y/o male	Rash, arthralgias, abdominal pain	3 months	2 mg/kg/day for 18 months (still active and off steroids for last 6 months)	Prednisone 1 mg/kg/day and taper over 14 months	N/A
Patient 5	9 y/o male	Rash, abdominal pain, renal disease	Intermittent disease over 4 years	1 mg/kg/day for 21 months	Prednisone 0.5 mg/kg/day and taper over 16 months	3.5 years
Patient 6	8 y/o female	Rash, arthralgias, abdominal pain	4 months	1 mg/kg/day for 13 months	Prednisone 0.5 mg/kg/day and taper over 6 months	20 months

## Case presentation

Patient 1 is a 7-year-old Caucasian female with HSP who initially presented with a rash on her lower and upper extremities. She was treated with 5 days of steroids. Three weeks later, she developed non-bloody emesis with a relapse of the rash on her face, upper and lower extremities and torso, leading to treatment with 1 mg/kg/day of prednisone. Three days later, she developed drainage of her lower extremity lesions and hematemesis. She was admitted and treated with IV methylprednisolone 2 mg/kg/day and IV fluids. On the fourth hospital day, she developed bloody stool after a few days of intermittent abdominal pain. An abdominal ultrasound showed small bowel intussusception, which was reduced with an air contrast enema. During her 21 days of hospitalization, she intermittently had abdominal pain and emesis after solid food re-introduction. Repeated attempts to taper oral glucocorticoids resulted in the relapse of her symptoms, leading to the use of IV methylprednisolone 1 mg/kg/day through a PICC. She received one dose of intravenous immunoglobulin (IVIG) 2 g/kg and was started on azathioprine 2 mg/kg/day as long-term maintanance therapy. Her laboratory evaluation was remarkable for an anti-nuclear antigen (ANA) of 1:2560 titer with negative anti-neutrophil cytoplasmic antibody (ANCA), anti-double stranded DNA (dsDNA), and extractable nuclear antigens (ENA). She had normal hemoglobin (Hb) 13.5 g/L, complement levels, and creatinine (Cr) 0.4 mg/dl and no proteinuria or hematuria. Skin biopsy was not obtained. Her symptoms gradually improved and she was discharged on a methylprednisolone taper that was discontinued 6 weeks after discharge. Her rash and mild abdominal symptoms persisted for 3 months post-discharge. She was continued on azathioprine for a total of 11 months, and 15 months after medication discontinuation, she remained asymptomatic with unremarkable continuous laboratory screening for an alternative diagnosis, such as systemic lupus erythematosus (SLE).

Patient 2 is an 11-year-old Caucasian female who presented with palpable purpura, abdominal pain, and arthritis. She was initially treated with NSAIDs. Over the following 3 weeks, she developed multiple recurrences of her rash affecting her upper and lower extremities and lower abdomen, as well as a painful lesion of her right sole that prevented her from walking. She was admitted and received 2 days of IV methylprednisolone 5 mg/kg q6 h for 5 doses. Due to the prolonged duration and severity of her rash, she was discharged on a prednisone taper of 0.5 mg/kg/day over 6 weeks, and started on methotrexate of 10 mg/m^2^ BSA. Skin biopsy was not obtained. Six months later, methotrexate was switched to azathioprine 1 mg/kg/day due to recurrent purpura. Her symptoms subsided, and within 2 months, control of her rash was achieved. Azathioprine was discontinued after 7 months, after which she remained asymptomatic without a rash. Laboratory testing revealed negative ANCA, sedimentation rate (ESR) of 18 mm/h, C-reactive protein (CRP) of 1.9 mg/L, Cr 0.5 mg/dl, Hb 12 g/dl, and an unremarkable urinalysis.

Patient 3 is an 8-year-old Caucasian female who presented with a purpuric rash of her lower extremities and arthritis of her ankles. Her symptoms subsided without the use of steroids, but she had persistent arthralgias. Four months after the initial episode, following a febrile illness, she developed a recurrence of her characteristic rash and worsening joint symptoms. She was started on a prednisone taper from an initial dose of 0.5 mg/kg/day but had recurrence of her joint symptoms during tapering. Her prednisone dose was increased to the initial dose of 0.5 mg/kg/day, and azathioprine 1 mg/kg/day was added. She discontinued prednisone 6 weeks later and continued to be asymptomatic, with the exception of a single episode of a breakthrough rash 4 months later which resolved with a 5 day course of prednisone. Over the following several months, she remained asymptomatic and azathioprine was discontinued 7 months after her last relapse, for a total of 13 months in total of azathioprine treatment. Laboratory testing revealed negative ANA, Hb 11 g/dl, ESR 9 mm/h, Cr 0.4 mg/dl, and no proteinuria or hematuria. Skin biopsy was not thought necessary to confirm the diagnosis.

Patient 4 is a 9-year-old Caucasian male diagnosed with HSP six weeks prior to presentation. He initially had partial improvement on prednisone 1 mg/kg/day but then developed worsening symptoms with his prednisone wean. His palpable purpuric rash was most prominent on his bilateral distal lower extremities but was also present mildly on his buttocks, abdomen, and arms. He complained of arthralgias, testicular pain, and mild abdominal pain with normal bowel movements without hematochezia, mucous or signs of intussusception. Prednisone was increased back to 1 mg/kg/day with improvement of symptoms. A second but slower prednisone taper resulted in the return/worsening of symptoms again at 0.5 mg/kg/day. Azathioprine was added 3 months after disease onset, with successful tapering off of prednisone and no return of symptoms for the last 6 months. Laboratory evaluation showed ESR 11, CRP 0.8 mg/dL, urinalysis without proteinuria or hematuria, Cr 0.44 mg/dl, Hb 13.5 g/dL, normal immunoglobulin A (IgA), normal complement levels, and negative ANA, anti-SSA, anti-SSB, ANCA, rheumatoid factor (RF), and anti-cyclic citrullinated peptide (CCP) antibodies. Skin biopsy was not obtained.

Patient 5 is a 9 year old Asian male with recurrent HSP over 4 years with manifestations of palpable purpura, abdominal pain with hematochezia but without intussusception, intermittent mild hematuria and proteinuria, and testicular pain with swelling. Laboratory evaluation was remarkable for ESR 23, CRP 8.3 mg/L, urinalysis without proteinuria but with brief intermittent hematuria, Cr 0.4, Hb 13.4 g/dL, normal IgA and complement levels, and negative ANA and ANCA antibodies. Skin biopsy was suggestive of leucocytoclastic vasculitis with IgA deposition typical of HSP. Renal biopsy was not performed. Azathioprine 1 mg/kg/day was added to prednisone 0.5 mg/kg/day due to the recurrent nature of his symptoms and the lack of a sustained response to glucocorticoids. The addition of azathioprine resulted in dramatic improvement of all symptoms, and tapering of prednisone was successful. Azathioprine was used for 21 months total prior to discontinuation, and the patient has currently sustained remission for 3.5 years off azathioprine and glucocorticoids.

Patient 6 is an 8-year-old Caucasian female with HSP diagnosed 3 months prior to presentation, with manifestations of palpable purpura, abdominal pain without hematochezia or intussusception, and mild lower extremity arthralgias with morning stiffness. Laboratory evaluation was remarkable for ESR 4, CRP 1.4 mg/L, urinalysis without proteinuria or hematuria, Cr 0.5, Hb 12.3 g/dL, normal IgA and complement levels, and negative ANA and ANCA antibodies. Skin biopsy was not obtained. Her symptoms were responsive to prednisone 0.5 mg/kg/day, but due to recurrence of symptoms during the glucocorticoid taper, azathioprine 1 mg/kg/day was added with rapid amelioration her symptoms. This allowed successful tapering off of prednisone. Azathioprine was used for 13 months prior to discontinuation, and the patient has sustained remission for 20 months off azathioprine and glucocorticoids.

## Conclusions

In this case series, we describe 6 patients meeting the classification criteria for HSP [12], who developed prolonged and relapsing symptoms and were successfully treated with azathioprine. None of these patients had classic renal involvement. The duration of treatment with azathioprine ranged from 7 to 21 months without a relapse of disease after discontinuing treatment.

The course of HSP is usually benign and self-limited, with only 1/3 of patients having recurrent disease. Since the disease is usually self-limited, typically no treatment is warranted. In the absence of renal disease, the initial episode usually lasts approximately 1 month [1]. Recurrence of disease occurs most commonly in patients with nephritis or more severe symptoms at presentation [13]. Treatment with glucocorticoids offers quick symptomatic relief and shortens the severity and duration of gastrointestinal, joint, and cutaneous symptoms [1, 14] Steroids are often used successfully in patients with limited oral intake due to their abdominal symptoms, a painful rash, or difficulty ambulating. [2]. However, treatment with glucocorticoids is controversial, as they do not appear to alter or improve the final outcome, including renal disease [15]. There are currently no established evidence-based recommendations for glucocorticoid use in HSP, although they are often used in clinical practice.

Management options for recalcitrant disease that is poorly responsive to steroids or is steroid-dependent have not been defined, and only a few case series of patients managed with other medications have been reported [3–11]. Azathioprine has been used with corticosteroids or other agents in cases of HSP nephritis (Table 2). Results were encouraging as this combination seemed to improve the clinical course of the nephritis, but randomized controlled trials (RCTs) are needed [16–21]. Only one case of hemorrhagic bullous cutaneous HSP successfully treated with azathioprine has been reported in the literature [11]. In addition, azathioprine has been used in cases of cutaneous leukocytoclastic vasculitis with IgA deposition that histologically resembles HSP [22]. Expert opinion favors the use of colchicine or dapsone as first line treatment for cutaneous leucocytoclastic vasculitis [23–25]. Efficacy of those agents is questionable, as the recommendations are based on case reports, while a randomized controlled trial failed to show efficacy of colchicine [26]. Azathioprine is recommended as second line treatment by most authors for the treatment of cutaneous leucocytoclastic vasculitis [23–25].

**Table 2:** Summary of studies and case reports identified by systemic literature search. We searched the Medline indexed literature after 1996 combining the words azathioprine, purpura, Schoenlein-Henoch and further searched the reference lists of identified articles for additional papers. We restricted results to English language published literature.

Author (year)	Number of patients	Reason for azathioprine use	Treatment used	Duration of study	Conclusions
Bergstein (1998) [16]	21	Crescentic HSP nephritis	Azathioprine and steroids	9–24 months and average follow up time of 32 months	Azathioprine is effective in reducing hematuria, proteinuria, serum creatinine and increasing creatine clearence in 19/21. Two patients progressed to ESKD
Foster (2000) [17]	17	Severe HSP nephritis	Azathioprine and prednisone	Azathioprine 1–4 mg/kg and prednisone for 46.7 +/− 19 weeks compared to historical controls treated in various ways	No progression of renal lesions after treatment initiation. Favorable outcome compared to historical control group.
Singh (2002) [18]	9	Severe HSP nephritis	Azathioprine and steroids	Azathioprine treatment of a mean of 14.7 months and steroids for 12.1 months, 4.7 years mean follow up	Mean resolution of proteinuria and hematuria 6.5 and 6.6 months respectively
Shin (2005) [19]	20	Severe HSP nephritis	Azathioprine and steroids versus steroids alone	Azathioprine for 8 months, steroids median of 1.7 years, median follow up 4.8 years	Azathioprine and steroids ameliorate histopathological features and improve the clinical course of severe HSP nephritis. No significant differences in hematuria and proteinuria between the groups.
Shenoy (2007) [20]	27	HSP nephritis grade IIIb or greater	Induction with steroids and CYC for 8–12 weeks followed by maintanance with azathioprine and steroids	Azathioprine for a mean of 12 months, 6.9 y mean follow up period,	37 % complete recovery, 40.7 % persistent proteinuria, 7.4 proteinuria and HTN, 14.8 % ESKD. Poor outcome on those being older at presentation and having crescents/heavy proteinuria 6 months post-diagnosis
Trapani (2010) [13]	1	Hemorrhagic bullous cutaneous involvement	Azathioprine 2.5 mg/kg with steroids	Follow up 5 months	Symptom resolution
Ninchoji (2011) [21]	32	Moderate or severe HSP nephritis	Azathioprine 2 mg/kg, steroids and anticoagulants for modarate severe and severe nephritis in comparison to ACE-I or ARB for moderate severe nephritis	Combination therapy including azathioprine for 6 months	90 % resolution of moderate HSP nephritis by 14.9 months and 90 % resolution of severe nephritis by 11.1 months

*ACE-I* angiotensin converting enzyme inhibitor, *ARB* angiotensin receptor blocker, *CYC* cyclophosphamide, *ESKD* end stage kidney disease, *HSP* Henoch-Schönlein purpura

Toxicity to the GI tract (elevation of liver enzymes, anorexia, nausea, vomiting) and the theoretical increased risk of malignancy are the main concerns of azathioprine use. The idiosyncratic arrest of granulocyte maturation and subsequent risk of infection may be reduced by adjusting the dose of azathioprine based on the measurement of thiopurine S-methyltranferase levels (TPMT), whose genetic variation has been related to the observed bone marrow toxicity [27]. No adverse events were reported in this case series, and safety monitoring included a complete blood count (CBC) and complete metabolic panel (CMP) to evaluate for liver toxicity or blood cell dyscrasias.

HSP is usually a clinical diagnosis, therefore, a skin biopsy is rarely obtained and usually no laboratory testing is necessary to establish a diagnosis [12, 28]. However, chronic or recurrent HSP symptoms should always raise concern for alternative causes of cutaneous small vessel vasculitis, such as ANCA-associated vasculitis or SLE [29, 30]. In addition, ulcerative colitis has been misdiagnosed as HSP [31], while Familial Mediterannean Fever (FMF) is also in the differential diagnosis. Frequently FMF patients develop HSP and a genetic association between FMF and HSP has been shown [32, 33]. Laboratory tests such as ANA, anti-dsDNA or ANCA antibodies are usually negative in HSP, and their positivity may be suggestive of another diagnosis. In these cases, careful clinical and laboratory diagnostic evaluation, including a skin biopsy, is warranted [1]. In this series a skin biopsy was obtained only in patient 5. In the other cases the appearance of the rash, the concomitant symptoms and laboratory testing were strongly supportive of a diagnosis of HSP, making biopsy unnecessary.

While HSP is typically a self-limited disease, our cases all demonstrated persistent or recurrent symptoms. It is unlikely the resolution of symptoms in these patients was simply due to the natural course of the disease and was more likely due to the effects of azathioprine. Azathioprine appeared to be an effective steroid-sparing medication, allowing all steroid-dependent patients to be successfully tapered off of steroids. No official guideline is available for treatment duration, and this case series does not establish the optimal duration of azathioprine treatment. However, once symptoms are controlled, it appears that discontinuation of therapy may be attempted empirically after complete disease remission has been achieved for 6–15 months.

Our experience with the use of azathioprine has also been favorable in 4 additional cases not reported here. These include 2 cases with persistent skin and renal disease responding to treatment but without enough follow up time, one case of relapsing skin disease responsive to treatment with azathioprine but without significant follow up time to be reported, and one patient with relapsing skin disease and renal involvement in which azathioprine successfully achieved remission. Only in one case with persistent skin and renal involvement did azathioprine 1 mg/kg fail to achieve renal symptom control and was discontinued within 2 months.

In summary, this case series demonstrates improved symptom control, steroid-sparing effect, and induction of remission with azathioprine in patients with recalcitrant HSP without renal disease. No patient had any adverse events associated with azathioprine therapy. Randomized controlled trials comparing the different therapeutic options for these type of patients are needed in the future.

## Abbreviations

ANA, antinuclear antibodies; ANCA, anti neutrophil cytoplasmic antibodies; CBC, complete blood count; Cr, creatinine; CRP, c-reactive protein; dsDNA, double stranded DNA; ENA, extractable nuclear antigen; ESR sedimentation rate; Hb, hemoglobin; HSP, Henoch-Schönlein purpura

## References

[1] Saulsbury FT (1999). Henoch-Schönlein purpura in children. Report of 100 patients and review of the literature. Medicine (Baltimore).

[2] Ronkainen J, Koskimies O, Ala-Houhala M, Antikainen M, Merenmies J, Rajantie J, Ormälä T, Turtinen J, Nuutinen M (2006). Early prednisone therapy in Henoch-Schönlein purpura: a randomized, double-blind, placebo-controlled trial. J Pediatr.

[3] Allali S, Fraitag S, Terrier B, Bodemer C, Chalumeau M (2016). Efficacy of colchicine in a child with relapsing bullous Henoch-Schönlein purpura. Eur J Pediatr.

[4] Saulsbury FT (2009). Successful treatment of prolonged Henoch-Schönlein purpura with colchicine. Clin Pediatr (Phila).

[5] Iqbal H, Evans A (2005). Dapsone therapy for Henoch-Schönlein purpura: a case series. Arch Dis Child.

[6] Donnithorne KJ, Atkinson TP, Hinze CH, Nogueira JB, Saeed SA, Askenazi DJ, Beukelman T, Cron RQ (2009). Rituximab therapy for severe refractory chronic Henoch-Schönlein purpura. J Pediatr.

[7] Rettig P, Cron RQ (2003). Methotrexate used as a steroid-sparing agent in non-renal chronic Henoch-Schönlein purpura. Clin Exp Rheumatol.

[8] Padeh S, Passwell JH (2000). Successful treatment of chronic Henoch-Schonlein purpura with colchicine and aspirin. Isr Med Assoc J.

[9] Martin S, Cramer CH, Heikenen J, Gitomer JJ (2006). Gastrointestinal symptoms of Henoch-Schönlein purpura treated with mycophenolate mofetil. J Pediatr Gastroenterol Nutr.

[10] Pindi Sala T, Michot J-M, Snanoudj R, Dollat M, Estève E, Marie B, Taoufik Y, Delfraissy J-F, Lazure T, Lambotte O (2014). Successful outcome of a corticodependent henoch-schönlein purpura adult with rituximab. Case Rep Med.

[11] Trapani S, Mariotti P, Resti M, Nappini L, de Martino M, Falcini F (2010). Severe hemorrhagic bullous lesions in Henoch Schonlein purpura: three pediatric cases and review of the literature. Rheumatol Int.

[12] Ozen S, Pistorio A, Iusan SM, Bakkaloglu A, Herlin T, Brik R, Buoncompagni A, Lazar C, Bilge I, Uziel Y, Rigante D, Cantarini L, Hilario MO, Silva CA, Alegria M, Norambuena X, Belot A, Berkun Y, Estrella AI, Olivieri AN, Alpigiani MG, Rumba I, Sztajnbok F, Tambic-Bukovac L, Breda L, Al-Mayouf S, Mihaylova D, Chasnyk V, Sengler C, Klein-Gitelman M (2008). EULAR/PRINTO/PRES criteria for Henoch-Schönlein purpura, childhood polyarteritis nodosa, childhood Wegener granulomatosis and childhood Takayasu arteritis: Ankara, Part II: Final classification criteria. Ann Rheum Dis.

[13] Trapani S, Micheli A, Grisolia F, Resti M, Chiappini E, Falcini F, De Martino M (2005). Henoch Schonlein purpura in childhood: epidemiological and clinical analysis of 150 cases over a 5-year period and review of literature. Semin Arthritis Rheum.

[14] Weiss PF, Klink AJ, Localio R, Hall M, Hexem K, Burnham JM, Keren R, Feudtner C (2010). Corticosteroids may improve clinical outcomes during hospitalization for Henoch-Schönlein purpura. Pediatrics.

[15] Hahn D, Hodson EM, Willis NS, Craig JC (1996). Cochrane Database of Systematic Reviews.

[16] Bergstein J, Leiser J, Andreoli SP (1998). Response of crescentic Henoch-Schoenlein purpura nephritis to corticosteroid and azathioprine therapy. Clin Nephrol.

[17] Foster BJ, Bernard C, Drummond KN, Sharma AK (2000). Effective therapy for severe Henoch-Schonlein purpura nephritis with prednisone and azathioprine: a clinical and histopathologic study. J Pediatr.

[18] Singh S, Devidayal, Kumar L, Joshi K, Minz RW, Datta U (2002). Severe Henoch-Schönlein nephritis: resolution with azathioprine and steroids. Rheumatol Int.

[19] Shin JI, Park JM, Shin YH, Kim JH, Lee JS, Kim PK, Jeong HJ (2005). Can azathioprine and steroids alter the progression of severe Henoch-Schönlein nephritis in children?. Pediatr Nephrol.

[20] Shenoy M, Bradbury MG, Lewis MA, Webb NJA (2007). Outcome of Henoch-Schönlein purpura nephritis treated with long-term immunosuppression. Pediatr Nephrol.

[21] Ninchoji T, Kaito H, Nozu K, Hashimura Y, Kanda K, Kamioka I, Shima Y, Hamahira K, Nakanishi K, Tanaka R, Yoshikawa N, Iijima K, Matsuo M (2011). Treatment strategies for Henoch-Schönlein purpura nephritis by histological and clinical severity. Pediatr Nephrol.

[22] Callen JP, Spencer LV, Burruss JB, Holtman J (1991). Azathioprine. An effective, corticosteroid-sparing therapy for patients with recalcitrant cutaneous lupus erythematosus or with recalcitrant cutaneous leukocytoclastic vasculitis. Arch Dermatol.

[23] Carlson JA, Cavaliere LF, Grant-Kels JM (2006). Cutaneous vasculitis: diagnosis and management. Clin Dermatol.

[24] Goeser MR, Laniosz V, Wetter DA (2014). A practical approach to the diagnosis, evaluation, and management of cutaneous small-vessel vasculitis. Am J Clin Dermatol.

[25] Micheletti RG, Werth VP (2015). Small vessel vasculitis of the skin. Rheum Dis Clin North Am.

[26] Sais G, Vidaller A, Jucglà A, Gallardo F, Peyrí J (1995). Colchicine in the treatment of cutaneous leukocytoclastic vasculitis. Results of a prospective, randomized controlled trial. Arch Dermatol.

[27] Belmont MH (2015). Pharmacology and side effects of azathioprine when used in rheumatic diseases.

[28] Ozen S, Ruperto N, Dillon MJ, Bagga A, Barron K, Davin JC, Kawasaki T, Lindsley C, Petty RE, Prieur AM, Ravelli A, Woo P (2006). EULAR/PReS endorsed consensus criteria for the classification of childhood vasculitides. Ann Rheum Dis.

[29] Wright AC, Gibson LE, Davis DMR (2015). Cutaneous manifestations of pediatric granulomatosis with polyangiitis: a clinicopathologic and immunopathologic analysis. J Am Acad Dermatol.

[30] Ramos-Casals M, Nardi N, Lagrutta M, Brito-Zerón P, Bové A, Delgado G, Cervera R, Ingelmo M, Font J (2006). Vasculitis in Systemic Lupus Erythematosus. Medicine (Baltimore).

[31] de Oliveira GT, Martins SS, Deboni M, Picciarelli P, Campos LMA, Jesus AA, Koda YKL, Silva CA (2013). Cutaneous vasculitis in ulcerative colitis mimicking Henoch-Schönlein purpura. J Crohns Colitis.

[32] Gershoni-Baruch R, Broza Y, Brik R (2003). Prevalence and significance of mutations in the familial Mediterranean fever gene in Henoch-Schönlein purpura. J Pediatr.

[33] Tekin M, Yalçinkaya F, Tümer N, Akar N, Misirlioğlu M, Cakar N (2000). Clinical, laboratory and molecular characteristics of children with Familial Mediterranean Fever-associated vasculitis. Acta Paediatr.

